# A New Oxadiazole-Based Topsentin Derivative Modulates Cyclin-Dependent Kinase 1 Expression and Exerts Cytotoxic Effects on Pancreatic Cancer Cells

**DOI:** 10.3390/molecules27010019

**Published:** 2021-12-21

**Authors:** Camilla Pecoraro, Barbara Parrino, Stella Cascioferro, Adrian Puerta, Amir Avan, Godefridus J. Peters, Patrizia Diana, Elisa Giovannetti, Daniela Carbone

**Affiliations:** 1 Dipartimento di Scienze e Tecnologie Biologiche Chimiche e Farmaceutiche (STEBICEF), Università degli Studi di Palermo, Via Archirafi 32, 90123 Palermo, Italy; camilla.pecoraro@unipa.it (C.P.); barbara.parrino@unipa.it (B.P.); stellamaria.cascioferro@unipa.it (S.C.); patrizia.diana@unipa.it (P.D.); 2 Department of Medical Oncology, Cancer Center Amsterdam, Amsterdam UMC, VU University Medical Center (VUmc), De Boelelaan 1117, 1081 HV Amsterdam, The Netherlands; apuertaa@ull.edu.es (A.P.); AvanA@mums.ac.ir (A.A.); gj.peters@amsterdamumc.nl (G.J.P.); 3 BioLab, Instituto Universitario de Bio-Orgánica “Antonio González” (IUBO-AG), Universidad de La Laguna, c/Astrofísico Francisco Sánchez 2, 38206 La Laguna, Spain; 4 Metabolic Syndrome Research Center, Mashhad University of Medical Science, Mashhad 91886-17871, Iran; 5 Department of Biochemistry, Medical University of Gdansk, 80-210 Gdansk, Poland; 6 Cancer Pharmacology Laboratory, Fondazione Pisana per la Scienza, Via Ferruccio Giovannini 13, San Giuliano Terme, 56017 Pisa, Italy

**Keywords:** 1,2,4-oxadiazole, marine alkaloids, topsentin, CDK1 inhibitor, pancreatic cancer, PDAC, antiproliferative, apoptosis

## Abstract

Pancreatic ductal adenocarcinoma (PDAC) is a highly lethal form of cancer characterized by drug resistance, urging new therapeutic strategies. In recent years, protein kinases have emerged as promising pharmacological targets for the treatment of several solid and hematological tumors. Interestingly, cyclin-dependent kinase 1 (CDK1) is overexpressed in PDAC tissues and has been correlated to the aggressive nature of these tumors because of its key role in cell cycle progression and resistance to the induction of apoptosis. For these reasons, CDK1 is one of the main causes of chemoresistance, representing a promising pharmacological target. In this study, we report the synthesis of new 1,2,4-oxadiazole compounds and evaluate their ability to inhibit the cell growth of PATU-T, Hs766T, and HPAF-II cell lines and a primary PDAC cell culture (PDAC3). Compound **6b** was the most active compound, with IC_50_ values ranging from 5.7 to 10.7 µM. Molecular docking of **6b** into the active site of CDK1 showed the ability of the compound to interact effectively with the adenosine triphosphate binding pocket. Therefore, we assessed its ability to induce apoptosis (which increased 1.5- and 2-fold in PATU-T and PDAC3 cells, respectively) and to inhibit CDK1 expression, which was reduced to 45% in Hs766T. Lastly, compound **6b** passed the ADME prediction, showing good pharmacokinetic parameters. These data demonstrate that **6b** displays cytotoxic activity, induces apoptosis, and targets CDK1, supporting further studies for the development of similar compounds against PDAC.

## 1. Introduction

Pancreatic ductal adenocarcinoma (PDAC), the most common type of pancreatic cancer, is a highly lethal form of cancer, for which surgery is the only curative treatment [1]. However, only a small percentage of PDACs are eligible for resection. Polychemotherapy regimens, such as FOLFIRINOX (folinic acid, fluorouracil, irinotecan, and oxaliplatin) or gemcitabine plus nab-paclitaxel, are the standard therapies for most PDAC patients but are characterized by a high rate of toxicity and modest survival benefit, since almost all PDAC patients become or are already drug resistant [2,3,4]. The causes of such drug resistance are many, and recent omics studies, including phosphoproteomics, have shown aberrations in key functional signaling that could hopefully be used to identify new therapeutic strategies [5].

In recent years, particular attention has been paid to protein kinases, which emerged as promising pharmacological targets given their role in regulating fundamental cellular processes. Since the first kinase inhibitor, imatinib, was approved for the treatment of chronic myeloid leukemia in 2001, the Food and Drug Administration has approved more than 50 small-molecule kinase inhibitors, of which the majority are tyrosine kinase inhibitors, while some are serine/threonine kinase inhibitors [6,7,8,9].

Cyclin-dependent kinase 1 (CDK1) is a serine/threonine kinase which plays a crucial role in regulating the cell cycle and has recently emerged as a promising target for the treatment of PDAC. Indeed, CDK1 overexpression was correlated with the progression of this type of cancer [10,11]. Under physiological conditions, CDK1 tightly regulates the progression of the cell cycle. However, abnormal expression of CDK1 determines cell replication even in case of DNA damage, resulting in the proliferation of cancer cells. Moreover, the activity of CDK1 is strongly regulated by the gene *TP53*, which is mutated in 70% of PDAC [12]. Together, these observations suggest the potential of CDK1 inhibition as a novel promising strategy to treat PDAC. They prompted us to synthesize new bioactive compounds against this target.

**Scheme 1:**
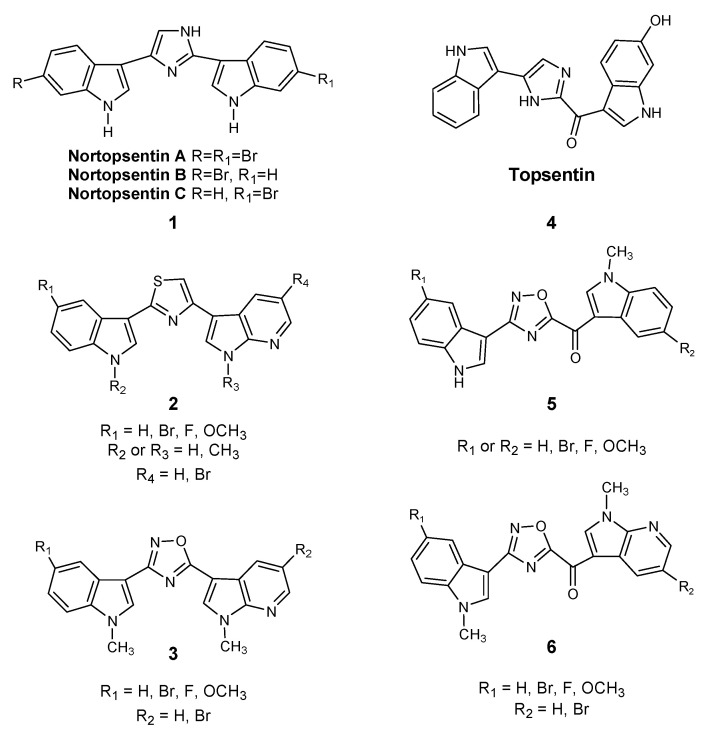
Chemical structures of nortopsentin **1** and topsentin **4** alkaloids and their derivatives **2**,**3** and **5**,**6**.

Considering the importance of marine microenvironments as an important resource of bioactive molecules containing different heterocyclic rings and different heteroatoms, our research group synthesized a number of small molecules obtained through the structural manipulation of nortopsentins **1**, natural bis-indolyl alkaloids isolated from deep-sea sponges (*Spongsorites ruetzleri*), which are characterized by significant antiproliferative activity against the P388 murine leukemia cell line [13]. In particular, we produced many derivatives in which the modification of nortopsentin involved the central imidazole ring, which was replaced by several five-membered heterocycles, while an indole moiety was substituted with an azaindole portion. These compounds had a significantly improved antiproliferative activity against a wide range of tumor cell lines, with half of the maximal inhibitory concentration (IC_50_) in the micro-submicromolar range [14,15]. In particular, thiazole nortopsentin derivatives **2**, determined CDK1 inhibition with IC_50_ values of 0.64–0.89 μM, which is comparable to the values reported for roscovitine and purvalanol A, two well-known CDK1 inhibitors. Moreover, a more recently synthesized indolyl-7-azaindolyl thiazole compound demonstrated its ability to reduce colorectal cancer stem cells (CR-CSCs), showing a synergistic effect with standard chemotherapy, such as oxaliplatin and 5-fluorouracil, as well as the ability of eradicating CR-CSCs when combined with the CHK1 inhibitor Rabusertinib [16]. Considering that, among nitrogen heterocycles, indole and oxadiazole rings are found in many molecules with significant biological activity, and especially antitumor activity [17,18,19], we also synthesized new 1,2,4-oxadiazole nortopsentin analogs **3**, which were screened for their antiproliferative activity [14]. Then, we further investigated the bis-indolyl marine alkaloid topsentin **4**, characterized by the presence of a carbonyl spacer group, which gives greater flexibility to the molecule to better adapt to the ATP-binding site of CDK1 and represents a hydrogen bonding acceptor, which could interact with the amino acid residues of the active site of CDK1. Topsentin was extracted from the sponge *Topsentia genitrix* and showed in vitro cytotoxic activity against P-388 murine tumor cells, with an IC_50_ of 8.8 μM, as well as at micromolar concentrations against several human cancer cell lines. Lastly, considering the promising results shown by [3-(1*H*-Indol-3-yl)-[1,2,4]oxadiazol-5-yl]-(1-methyl-1*H*-pyrrolo[2,3-*b*]pyridin-3-yl)-methanones **5** on PDAC cancer cell lines [20], we decided to synthesize a new series of topsentin analogs of the type **6** shown in Scheme 1, in which the imidazole central ring was replaced by 1,2,4-oxadiazole moiety, and one indole portion was converted into 7-azaindole ring (Scheme 1). In the present study, we also report the cytotoxic activity of these new derivatives against PDAC cells as well as their capability to inhibit CDK1 and to induce apoptosis.

## 2. Results and Discussion

### 2.1. Chemistry

**Scheme 2:**
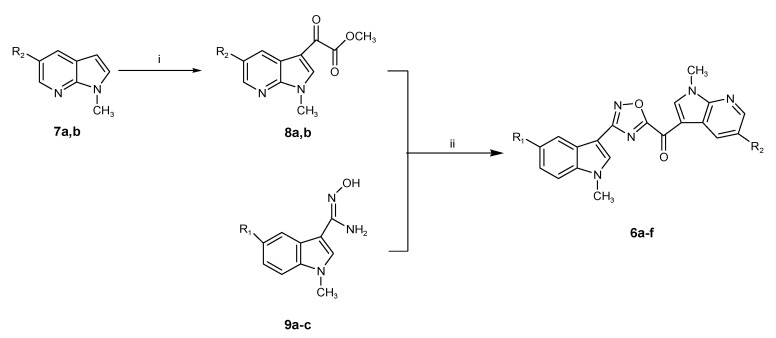
Synthesis of (1-methyl-1*H*-pyrrolo[2,3-*b*]pyridin-3-yl)-[3-(1-methyl-1*H*-indol-3-yl)-[1,2,4]oxadiazol-5-yl]-methanones (**6a–f**). Reagents and conditions: (i) oxalyl chloride, diethyl ether, r.t., overnight, then −65 °C, sodium methoxide solution 25 wt. % in methanol, r.t., 2 h, 62–73%; (ii) dimethylsulfoxide, r.t., 30 min. 60–85%.

A new series of new 7-azaindolyl oxadiazole compounds **6a–f** was efficiently synthesized as described in Scheme 2.

The key intermediates, (1-methyl-1*H*-pyrrolo[2,3-*b*]pyridine-3-yl)-oxo-acetic acid methyl esters **8a**,**b**, were prepared in good yield (62% and 73%, respectively) by keeping the corresponding methylindole precursor of the type **7a,b**, synthesized as previously described [14], with an excess of oxalyl chloride. The resulting non-isolated acetyl chloride intermediate was then converted in situ into methyl esters **8a,b** using a sodium methoxide solution 25 wt. % in methanol.

The latter compounds were subjected to a coupling reaction with carboxamidines **9a–c**, synthesized in turn from the corresponding 1-methyl-1*H*-indoles, converted to their carbonitriles, and had a successive reaction with hydroxylamine hydrochloride, as previously reported [21].

The azaindole substituent of the original oxo-acetic acid methyl ester is in position C(5), and the oxadiazole ring is obtained by means of the [4+1] synthetic route, as previously explained [21]. The coupling reaction, performed in anhydrous dimethylsulfoxide (DMSO) at r.t. for 30 min, proceeded without the isolation of an acylamidoxime intermediate, yielding, after purification by chromatography, the desired 7-azaindolyl oxadiazoles **6a–f** (Scheme 2) in yields ranging from 60% to 85% (Table 1).

### 2.2. Biological Studies

#### 2.2.1. Antiproliferative Activity of the New 1,2,4-Oxadiazole Compounds **6a–f** against PDAC3, PATU-T, Hs766T, and HPAF-II PDAC Cells

The in vitro antiproliferative activity of the new 7-azaindolyl oxadiazole compounds **6a–f** was initially evaluated by the sulforhodamine-B assay (SRB) against PATU-T immortalized PDAC cell line. All compounds were tested at three different concentrations of 50.0, 5.0, and 0.5 µM.

**Figure 1:**
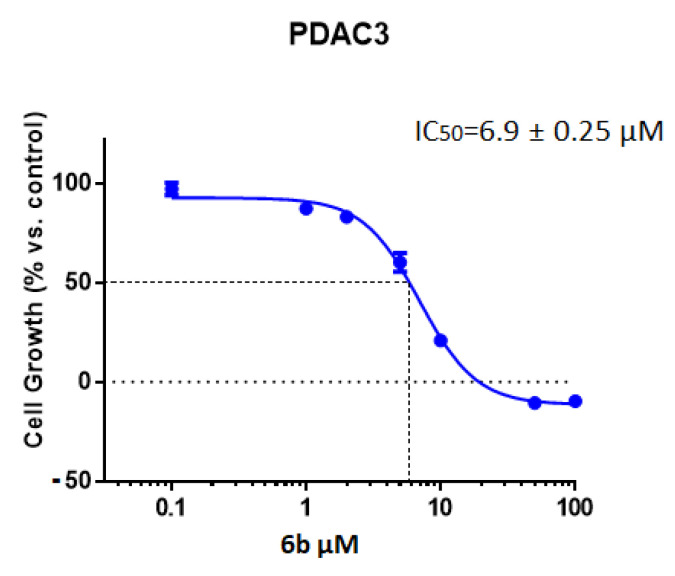
Representative growth inhibition curve of PDAC3 PDAC primary cells, treated for 72 h with the compound **6b**. Points: mean values obtained from three independent experiments; bars: SEM.

Among the tested compounds, derivative **6b** showed the highest potency, exhibiting an IC_50_ value of 10.7 µM, while other compounds showed minimal cytotoxic effect with IC_50s_ > 20 µM. In order to extend the antiproliferative evaluation of compound **6b** towards other pancreatic cells, we assessed the inhibition of cell growth in primary pancreatic cell lines including both the immortalized HPAF-II and Hs766T cancer cell lines and the primary culture PDAC3. Interestingly, compound **6b** was active against all these PDAC cells. The best result was observed against Hs766T, with an IC_50_ value of 5.7 µM. However, the compound **6b** was also able to inhibit the viability of PDAC3 and HPAF-II with IC_50_ values of 6.9 and 9.8 μM, respectively (Table 2). A representative curve of cell growth inhibition of **6b** in PDAC3 is reported in Figure 1. In parallel experiments we also evaluated the IC_50_ of the conventional anticancer drug gemcitabine, which was used as positive control and presented IC_50_ value below 1 µM. This result was in agreement with previous studies [22,23,24].

From structure activity relationship (SAR) analysis on compounds of type **5** and **6** (Scheme 1), we observed that the introduction of a nitrogen atom at position 7 of the indole moiety does not significantly improve the antiproliferative activity, as well as the presence of a methyl group at the indole N-atom. Conversely, in accordance with previous results concerning [3-(1*H*-indol-3-yl)-1,2,4-oxadiazol-5-yl](1-methyl-1*H*-indol-3-yl) methanones **5** [20], in type **6** derivatives the presence of a halogen atom, e.g., fluorine or bromine, at 5 position of the indole or 7-azaindole ring seems to be essential for the cytotoxic activity.

#### 2.2.2. Modulation of CDK1 Expression by ELISA

**Figure 2:**
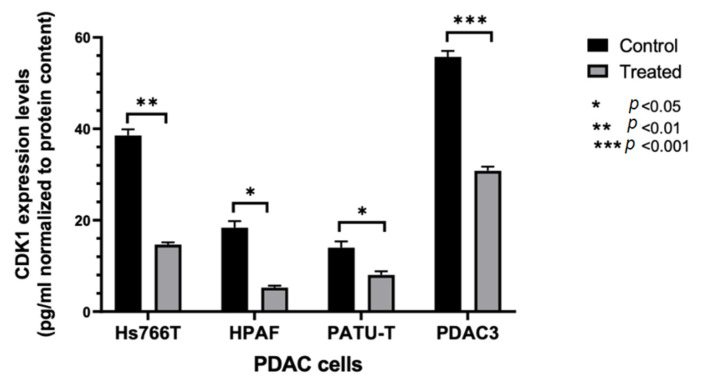
ELISA-based quantitation of CDK1 expression in PDAC cells following a 24 h exposure to DMSO (control) or to 10 µM of compound **6b**. CDK1 expression was quantified using Human cyclin-dependent kinase 1 (CDK1) ELISA Kit according to the manufacturer’s instructions. Columns: mean; bars: SEM (*n* = 3). *: *p* < 0.05, **: *p* < 0.01, ***: *p* < 0.001, significantly different compared with control.

Considering the promising results obtained with compound **6b**, further studies were carried out to investigate its mechanism of action. In order to evaluate whether compound **6b** was able to reduce CDK1 expression, a specific ELISA assay was performed in the three immortalized PDAC cell lines, i.e., Hs766T, HPAF-II, and PATU-T, as well as in the PDAC3 cells. These cell models were treated with 10 μM **6b**. As shown in Figure 2, we observed a reduction of CDK1 expression compared to control cells, supporting the potential role of the inhibition of CDK1 in the mechanism of action of this compound.

#### 2.2.3. Induction of Apoptosis

Considering that a number of previous studies reported that the inhibition of CDK1 activity contributes to the initiation of apoptotic process [25,26,27], we then evaluated the effect of **6b** on the induction of apoptosis in pancreatic cancer cells. For this purpose, we measured the externalization of the plasma membrane phosphatidylserine, a reliable marker of cell apoptosis, which was quantified by measurement of fluorescence of annexin V by spectrophotometric and microscopy assays.

**Figure 3:**
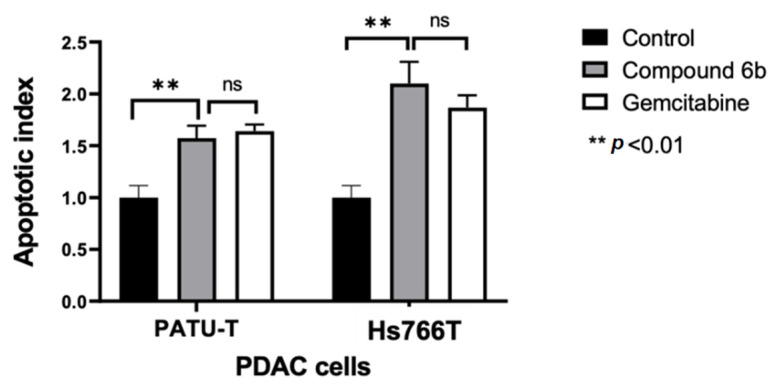
Effects of compound **6b** on apoptosis induction in PATU-T and Hs766T pancreatic cancer cells. The apoptotic index was calculated by evaluating the annexin V-FITC fold change compared with control cells after 24 h treatment. Gemcitabine was used as a positive control. Columns: mean; bars: SEM (*n* = 3). **: *p* < 0.01, significantly different compared with control; ns: not significant.

These experiments were performed on the most sensitive and most resistant models, i.e., Hs766T and PATU-T cells. After 24 h of treatment with 2*IC_50_ of **6b**, a significant increase in the portion of apoptotic cells was observed. In particular, we noticed that the percentages of apoptotic Hs766T and PATU-T cells were comparable to the number of cells that underwent apoptosis after treatment with gemcitabine (at 2*IC_50_), which is a standard drug for the treatment of PDAC (Figure 3).

#### 2.2.4. Molecular Modeling

**Figure 4:**
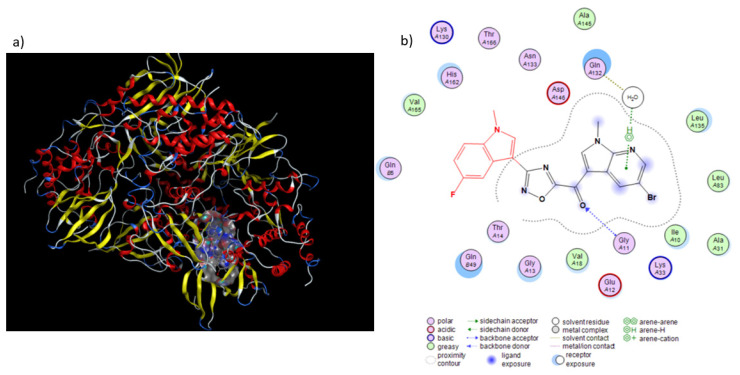
Proposed binding mode of compounds **6b** with CDK1 (PDB ID: 4YC6). (**a**) Surface representation of **6b** in CDK1 binding pocket. (**b**) H-bonds between the carbonyl group of our compound with the peptide nitrogen of Gly11 residue is shown with blue dashed arrow, while the water-mediated interaction between the nitrogen atom of the 7-azaindole portion adjacent to the oxadiazole ring and the Gln132 residue is depicted with green dashed lines.

A potential binding mode for the most active compound **6b** within the ATP binding site of CDK1 (PDB ID: 4YC6) is depicted in Figure 4. **6b** is placed in a nucleotide pocket, establishing a hydrogen bond through the carbonyl group with the backbone of Gly11 residue.

Moreover, the nitrogen group of 7-azaindole moiety accepts a hydrogen bond from a water molecule interacting with Gln132, a residue well known to constitute the DFG motif and to establish water-mediated interaction with H-bond acceptors of CDK1 inhibitors (Figure 4) [28].

The molecular docking scores of compound **6b** was found to be −6.999 Kcal/mol, indicating efficient binding to the active site of CDK1.

#### 2.2.5. ADME Prediction

In order to evaluate and predict the fate of compound **6b** within the human body, ADME and pharmacokinetic predictions were carried out using a freely available in silico ADME software [29]. Compound **6b**, as shown in Table 3, displayed promising physicochemical and pharmacokinetic parameters in ADME prediction studies, showing 3 H-bond acceptors, 0 H-bond donors, 3 rotatable bonds, and log *p* < 5. Moreover, our compound respects the Lipinski rule of 5, a feature making it a promising hit compound in the drug discovery field. Compound **6b** displayed a high gastrointestinal (GI) absorbance score but is not predicted to cross the blood brain barrier (BBB).

## 3. Materials and Methods

### 3.1. Chemistry

All melting points were taken on a Buchi-Tottoly capillary apparatus and were uncorrected. IR spectra were determined in bromoform using a Shimadzu FT/IR 8400S spectrophotometer. ^1^H and ^13^C NMR spectra were measured at 200 and 50 MHz, respectively, in DMSO-*d_6_* solution, using a Bruker Avance II series 200 MHz spectrometer. Chromatography was performed with a MERK silica gel 230–400 mesh ASTM column or a FLASH40i Biotage or with a Buchi Sepacore chromatography module (prepacked cartridge reference). Elementary analyses (C, H, N) were within ± 0.4% of the theoretical values.

#### 3.1.1. Synthesis of 1-methyl-1*H*-pyrrolo[2,3-*b*]pyridines (**7a,b**)

*t*-BuOK (0.38 g, 3.4 mmol) and TDA-1 (1 or 2 drops) were added to a cold solution of appropriate commercial 7-azaindoles (2.5 mmol) in anhydrous toluene (25 mL), at 0 °C. The reaction mixture was stirred at room temperature for 3 h, and then methyl iodide (2.5 mmol, 0.2 mL) was added at 0 °C. TLC analysis (cyclohexane/ethyl acetate 7:3) revealed that methylation was complete after 1 h. The solvent was evaporated under reduced pressure. The residue was treated with water, extracted with DCM (3 × 20 mL), dried (Na_2_SO_4_), evaporated, and purified by column chromatography using DCM/ethyl acetate (9/1) as the eluent, to give the desired product.

For 5-Bromo-1-methyl-1*H*-pyrrolo[2,3-*b*]pyridine (**7a**) and 1-Methyl-1*H*-pyrrolo[2,3-*b*]pyridine (**7b**), analytical and spectroscopic data were in accordance to those reported in literature [14].

#### 3.1.2. Synthesis of (1-methyl-1*H*-pyrrolo[2,3-*b*]pyridin-3-yl)-oxo-acetic Acid Methyl Esters (**8a,b**)

Oxalyl chloride (0.42 mL, 4.8 mmol) was added dropwise to a solution of appropriate 1-methyl-1*H*-pyrrolo[2,3-*b*]pyridine **7a,b** (1 g, 4.8 mmol) in dry diethyl ether (8.5 mL) under a nitrogen atmosphere at 0 °C. The reaction mixture was stirred overnight at room temperature. The solution was cooled to −65 °C using an acetone bath with an immersion cooler, before adding a sodium methoxide solution, 25 wt. % in methanol (11 mL, 10.8 mmol, 2.3 eq.) dropwise. The reaction mixture was heated up to room temperature and stirred for 2 h. The reaction was quenched with brine (1 mL) and water (1 mL), and the obtained precipitate was filtered off. Upon filtering, the isolated solid was dried under high vacuum overnight.

*(5-Bromo-1-methyl-1H-pyrrolo[2,3-b]pyridin-3-yl)-oxo-acetic acid methyl ester* (**8a**)
  Yield: 62%; light yellow solid; mp: 140.5–141.5 °C; IR (cm^−1^): 1731 (CO), 1653 (CO); ^1^H-NMR (200 MHz, DMSO-*d_6_*) δ: 3.91 (3H, s, CH_3_), 3.92 (3H, s, OCH_3_), 8.54 (1H, d, *J* = 2.2 Hz, H-4), 8.57 (1H, d, *J* = 2.2 Hz, H-6), 8.80 (1H, s, H-2); ^13^C NMR (50 MHz, DMSO-*d_6_*) δ: 32.0 (q), 52.8 (q), 109.2 (s), 114.7 (s), 131.4 (d), 143.0 (d), 145.1 (d), 146.6 (s), 162.6 (s), 176.8 (s), 177.6 (s); *Anal.* calculated for C_11_H_9_BrN_2_O_3_ (MW: 297.10): C, 44.47; H, 3.05; N, 9.43%. Found: C, 44.58; H, 3.24; N, 9.65%.

*(1-Methyl-1H-pyrrolo[2,3-b]pyridin-3-yl)-oxo-acetic acid methyl ester* (**8b**)
  Yield: 73%; white solid; mp: 97.8–98.8 °C; IR (cm^−1^): 1729 (CO), 1650 (CO); ^1H-NMR^ (200 MHz, DMSO-*d_6_*) δ: 3.91 (3H, s, CH_3_), 3.93 (3H, s, OCH_3_), 7.38 (1H, dd, *J* = 7.8, 4.8 Hz, H-4), 8.44–8.51 (2H, m, H-5 and H-6), 8.74 (1H, s, H-2); ^13^C NMR (50 MHz, DMSO-*d_6_*) δ: 31.8 (q), 52.7 (q), 109.7 (s), 118.3 (s), 119.3 (d), 129.8 (d), 141.8 (d), 144.9 (d) 148.1 (s), 163.1 (s), 176.8 (s), 178.0 (s); *Anal.* calculated for C_11_H_10_N_2_O_3_ (MW: 218.21): C, 60.55; H, 4.62; N, 12.84%. Found: C, 60.38; H, 4.78; N, 12.72%.

#### 3.1.3. Synthesis of (1-methyl-1*H*-pyrrolo[2,3-*b*]pyridine-3-yl)-[3-(1-methyl-1*H*-indol-3-yl)-[1,2,4]oxadiazol-5-yl]-methanones (**6a**–**f**)

A fine powder of sodium hydroxide (60 mg, 1.5 mmol) was quickly added to a solution of appropriate (1-methyl-1*H*-pyrrolo[2,3-*b*]pyridine-3-yl)-oxo-acetic acid methyl ester **8a,b** (446 mg, 1.5 mmol) and a suitable carboxamidine **9a–c** (190 mg, 1.0 mmol) in dry dimethylsulfoxide (DMSO) (2 mL). The resulting solution was allowed to stir at room temperature for 30 min. Then, water and ice were slowly added, and the obtained precipitate was filtered off. The residue was purified by column chromatography using cyclohexane/ethyl acetate (1/1) as the eluent.

*(5-Bromo-1-methyl-1H-pyrrolo[2,3-b]pyridin-3-yl)-[3-(1-methyl-1H-indol-3-yl)-[1,2,4]oxadiazol-5-yl]-methanone* (**6a**)
  Yield: 60%; yellow solid; mp: 258 °C (dec); IR (cm^−1^): 1630 (CO); ^1^H-NMR (200 MHz, DMSO-*d_6_*) δ: 3.90 (3H, s, CH_3_), 3.98 (3H, s, CH_3_), 7.21–7.33 (2H, m, H-5′ and H-6′), 7.56–7.60 (1H, m, H-7′), 7.99–8.06 (1H, m, H-4′), 8.37 (1H, s, H-2′), 8.56 (1H, d, *J* = 2.2 Hz, H-4), 8.68 (1H, d, *J* = 2.2 Hz, H-6), 9.24 (1H, s, H-2); *Anal.* calculated for C_20_H_14_BrN_5_O_2_ (MW: 436.26): C, 55.06; H, 3.23; N, 16.05%. Found: C, 55.18; H, 3.04; N, 15.89%.

*(5-Bromo-1-methyl-1H-pyrrolo[2,3-b]pyridin-3-yl)-[3-(5-fluoro-1-methyl-1H-indol-3-yl)-[1,2,4]oxadiazol-5-yl]-methanone* (**6b**)
  Yield: 67%; yellow solid; mp: 246 °C (dec); IR (cm^−1^): 1635 (CO); ^1^H-NMR (200 MHz, DMSO-*d_6_*) δ: 3.97 (3H, s, CH_3_), 4.04 (3H, s, CH_3_), 7.17–7.28 (1H, m, H-6′), 7.65–7.78 (2H, m, H-7′ and H-4′), 8.50 (1H, s, H-2′), 8.61 (1H, d, *J* = 1.8 Hz, H-4), 8.73 (1H, d, *J* = 1.8 Hz, H-6), 9.30 (1H, s, H-2); *Anal.* calculated for C_20_H_13_BrFN_5_O_2_ (MW: 454.25): C, 52.88; H, 2.88; N, 15.42%. Found: C, 55.80; H, 3.00; N, 15.55%.

*(5-Bromo-1-methyl-1H-pyrrolo[2,3-b]pyridin-3-yl)-[3-(5-methoxy-1-methyl-1H-indol-3-yl)-[1,2,4]oxadiazol-5-yl]-methanone* (**6c**)
  Yield: 85%; yellow solid; mp: 288 °C (dec); IR (cm^−1^): 1637 (CO); ^1^H-NMR (200 MHz, DMSO-*d_6_*) δ: 3.86 (3H, s, CH_3_), 3.92 (3H, s, CH_3_), 4.02 (3H, s, OCH_3_), 6.95–7.08 (1H, m, H-6′), 7.54–7.55 (2H, m, H-7′ and H-4′), 8.35 (1H, s, H-2′), 8.60 (1H, s, H-4), 8.72 (1H, s, H-6), 9.29 (1H, s, H-2); *Anal.* calculated for C_21_H_16_BrN_5_O_3_ (MW: 466.29): C, 54.09; H, 3.46; N, 15.02%. Found: C, 54.28; H, 3.30; N, 15.15%.

*(1-Methyl-1H-pyrrolo[2,3-b]pyridin-3-yl)-[3-(1-methyl-1H-indol-3-yl)-[1,2,4]oxadiazol-5-yl]-methanone* (**6d**)
  Yield: 77%; yellow solid; mp: 230 °C (dec); IR (cm^−1^): 1620 (CO); ^1^H-NMR (200 MHz, DMSO-*d_6_*) δ: 3.97 (3H, s, CH_3_), 4.05 (3H, s, CH_3_), 7.28–7.40 (2H, m, H-5′ and H-6′), 7.46 (1H, dd, *J* = 9.9, 4.8 Hz, H-4), 7.65 (1H, dd, *J* = 6.5, 2.1 Hz, H-7′), 8.11 (1H, dd, *J* = 6.2, 2.4 Hz, H-4′), 8.44 (1H, s, H-2′), 8.51 (1H, dd, *J* = 4.7, 1.3 Hz, H-6), 8.64 (1H, dd, *J* = 7.9, 1.3 Hz, H-5), 9.27 (1H, s, H-2); *Anal.* calculated for C_20_H_15_N_5_O_2_ (MW: 357.37): C, 67.22; H, 4.23; N, 19.60%. Found: C, 67.08; H, 4.04; N, 19.78%.

*(1-Methyl-1H-pyrrolo[2,3-b]pyridin-3-yl)-[3-(5-fluoro-1-methyl-1H-indol-3-yl)-[1,2,4]oxadiazol-5-yl]-methanone* (**6e**)
  Yield: 68%; yellow solid; mp: 257 °C (dec); IR (cm^−1^): 1638 (CO); ^1^H-NMR (200 MHz, DMSO-*d_6_*) δ: 3.98 (3H, s, CH_3_), 4.05 (3H, s, CH_3_), 7.18–7.28 (1H, m, H-6′), 7.42–7.50 (1H, m, H-4), 7.66–7.79 (2H, m, H-7′ and H-4′), 8.50 (1H, s, H-2′), 8.52–8.67 (2H, m, H-5 and H-6), 9.26 (1H, s, H-2); *Anal.* calculated for C_20_H_14_FN_5_O_2_ (MW: 375.36): C, 64.00; H, 3.76; N, 18.66%. Found: C, 64.20; H, 3.64; N, 18.50%.

*(1-Methyl-1H-pyrrolo[2,3-b]pyridin-3-yl)-[3-(5-methoxy-1-methyl-1H-indol-3-yl)-[1,2,4]oxadiazol-5-yl]-methanone* (**6f**)
  Yield: 80%; yellow solid; mp: 231 °C (dec); IR (cm^−1^): 1623 (CO); ^1^H-NMR (200 MHz, DMSO-*d_6_*) δ: 3.87 (3H, s, CH_3_), 3.92 (3H, s, CH_3_), 4.04 (3H, s, OCH_3_), 6.95–7.08 (1H, m, H-6′), 7.40–7.63 (3H, m, H-7′, H-4′ and H-4), 8.31–8.67 (3H, m, H-2′, H-5 and H-6), 9.29 (1H, s, H-2); *Anal.* calculated for C_21_H_17_N_5_O_3_ (MW: 387.39): C, 65.11; H, 4.42; N, 18.08%. Found: C, 65.28; H, 4.60; N, 18.25%.

### 3.2. Biology

#### 3.2.1. Drugs and Chemicals

The 1,2,4-oxadiazole compounds **6a–f** were synthesized at the Department of Pharmacy, University of Palermo (Palermo, Italy). The drugs were dissolved in DMSO. The medium, fetal bovine serum (FBS), penicillin (50 IU mL^−1^), and streptomycin (50 µg mL^−1^) were from Gibco (Gaithersburg, MD, USA). All other chemicals were from Sigma (Zwijndrecht, the Netherlands).

#### 3.2.2. Cell Cultures

HPAF-II, Hs766T, and PATU-T cell lines were purchased from the ATCC (American Type Culture Collection) (Manassas, VA, USA). The cell lines were tested for their authentication by STR–PCR (Short Tandem Repeat-Polymerase Chain Reaction). The primary cell line, PDAC3, was obtained from a patient undergoing pancreaticoduodenectomy, as described previously [30]. Additionally, all these cells were routinely tested for mycoplasma using PCR.

The cells were cultured in RPMI-1640 (Roswell Park Memorial Institute 1640) supplemented with 10% heat-inactivated FBS and 1% penicillin/streptomycin or in DMEM (Dulbecco’s Modified Eagle’s Medium), supplemented with 10% heat-inactivated FBS and 1% HEPES (4-(2-hydroxyethyl)-1-piperazineethanesulfonic acid). The cells were kept in a humidified atmosphere of 5% CO_2_ and 95% air at 37 °C and harvested with trypsin-EDTA (Ethylenediaminetetraacetic acid).

#### 3.2.3. Inhibition of Cell Growth

To evaluate the inhibitory effects of the 1,2,4-oxadiazole compounds **6a–f** on cell growth, we performed a Sulforhodamine-B (SRB) assay, as previously described [31,32]. Cells were seeded into 96-well flat-bottom plates in triplicate at a density of 3 × 10^3^ cells/well. Cells were incubated at 37 °C for 24 h to create a confluent monolayer, and then they were treated with 100 µL of increasing concentrations of the compounds dissolved in DMSO. After 72 h of treatment, the cells were fixed with 25 µL of 50% cold trichloroacetic acid and kept for at least 60 min at 4 °C. Then, the plates were washed gently with deionized water, dried at r.t. overnight, and stained with 50 µL of 0.4% SRB solution in 1% acetic acid for 15 min at r.t. The excess SRB stain was removed on dried tissues, and the plates were washed with 1% acetic acid and left to dry at r.t. overnight. The SRB was dissolved in 150 µL of tris(hydroxymethyl)aminomethane solution (pH = 8.8 (TRIS base)), and the optical density (OD) was detected at a wavelength of 490 nm and 540 nm. Cell growth inhibition was calculated as the percentage versus vehicle-treated cells (“negative control”), OD (corrected for OD before drug addiction). Finally, the half maximal inhibitory concentration (IC_50_) was calculated using a non-linear least squares curve fitting (GraphPad Prism 7, Intuitive Software for Science, San Diego, CA, USA).

#### 3.2.4. Enzyme-Linked Immunosorbent Assay (ELISA) for CDK1 Expression

The expression of CDK1 was detected and quantified using an Enzyme-Linked Immunosorbent Assay (ELISA, Human cyclin-dependent kinase 1 (CDK1) ELISA Kit, Catalog Number: MBS707090) according to the manufacturer’s protocol and our previous studies [33]. Volumes of 100 μL of the standard and the sample were added for each well, and the plates were incubated for 2 h at 37 °C. Then, the liquid was removed, and 100 μL of Biotin-antibody was added to each well, the plates were then incubated for 1 h at 37 °C. The medium was removed, and the plates were washed with Wash Buffer (200 µL). Subsequently, 100 μL of HRP-avidin was added to each well, and the plates were incubated for 1 h at 37 °C. The washing process was repeated five times. At the end, 90 µL of TMB Substrate was added to each well, and the plates were incubated for 30 min at 37 °C in the dark. Finally, 50 μL of stop solution was added to each well. The optical density of each well was determined using a microplate reader set to 450 nm.

#### 3.2.5. Apoptosis

Cells were seeded in 96-well plates (5 × 10^3^ cells/well) and, after one day, treated with drugs at the indicated concentrations for 24 h. At the end of the treatments, cells were fixed with 4% paraformaldehyde in PBS at r.t. for 30 min, then washed twice with PBS and stained with annexin V-FITC in a binding buffer (10 mM HEPES/NaOH pH 7.4, 140 mM NaCl, and 2.5 mM CaCl_2_) for 10 min at r.t. in the dark. Finally, cells were washed with binding buffer solution and fluorescence was measured using a multimode plate reader with excitation and emission filters at 485 nm and 535 nm, respectively. Parallel studies using the same method, counted the cells at the fluorescence microscopy and evaluated the apoptotic index. The values were normalized on cell proliferation using a crystal violet assay.

#### 3.2.6. Statistical Analysis

All the SRB and ELISA assays were carried out in triplicate and repeated at least three times, whereas the percentages of apoptotic cells were calculated taking into account three biological replicates. The data were evaluated using a GraphPad Prism (GraphPad Software, San Diego, CA, USA). Data were expressed as mean values ± SEM and analyzed using a Student’s *t*-test.

### 3.3. In Silico Studies

#### 3.3.1. Molecular Modelling and Docking

The molecular docking into CDK1 was performed for the compound **6b**. The CDK1 (PDB: 4YC6) X-ray crystal structure with a resolution of 2.60 Å, R-value 0.227 (observed), was obtained from the protein data bank [34]. The ligand **6b** was saved as a mol file, and the docking was performed using the Molecular Operating Environment (MOE) 2015.10.

#### 3.3.2. ADME Studies

The ADME predictions were performed using SwissADME prediction software. The number of H-bond donors, H-bond acceptors, and rotatable bonds, as well as the bioavailability, GI absorption, and BBB permeation were evaluated.

## 4. Conclusions

In the present study we efficiently synthesized a new series of 1,2,4-oxadiazole derivatives **6a–f** that showed promising antiproliferative activities in vitro against a series of PDAC cells.

PDAC is one of the most lethal forms of cancer, characterized by poor survival rates of 5 years, late diagnosis, and lack of effective treatment. The American Cancer Society estimated that 48,220 people will die of pancreatic cancer in the United States (US) in 2021 [35]. Due to the lack of specific symptoms, most patients are diagnosed at an advanced stage and cannot undergo surgical resection. The currently available therapeutic regimens include combinations of standard chemotherapy drugs, such as FOLFIRINOX [33,34]. However, PDACs are typically resistant to these treatments, and new therapeutic strategies are urgently needed.

Most PDACs harbor mutations linked to cell-cycle regulation [36], and *TP53* is one of the most frequent and relevant driver genes of pancreatic tumorigenesis. When the *TP53* gene is mutated, CDK1 is no longer inhibited and stimulates progression through the cell cycle [10]. In addition, recent studies have shown that CDK1 overexpression is associated with more advanced stages of significantly shorter survival of PDAC patients [37]. Thus, CDK1 inhibition seems a promising strategy for the treatment of PDAC, and there are multiple preclinical studies and clinical trials investigating the efficacy and tolerability of CDK1-inhibiting drugs.

Because of the key role of CDK1 in the regulation of apoptosis, we assessed the ability of compound **6b** to induce apoptosis, which proved to be comparable to that observed for gemcitabine. As one of the major hallmarks of PDAC is its resistance to apoptosis induction by gemcitabine [38], these results support future investigation of this compound as an excellent candidate for combination with gemcitabine.

In addition, ADME predictions demonstrated that compound **6b** possessed good pharmacokinetic properties, followed the Lipinski rule of five, and had a high level of gastrointestinal absorption, indicating that our compound could be formulated as an oral drug.

Overall, the results of cytotoxicity, modulation of CDK1 expression, and apoptosis obtained with compound **6b** support its role as an interesting hit compound for further chemical modification and biological analysis in order to discover new compounds for the treatment of patients with PDAC.

## Figures and Tables

**Table 1:** Yields of the synthesis of **6a–f**; (1-Methyl-1*H*-pyrrolo[2,3-*b*]pyridin-3-yl)-[3-(1-methyl-1*H*-indol-3-yl)-[1,2,4]oxadiazol-5-yl]-methanones.

Compound	R_1_	R_2_	Yield (%)
**6a**	H	Br	60%
**6b**	F	Br	67%
**6c**	OCH_3_	Br	85%
**6d**	H	H	77%
**6e**	F	H	68%
**6f**	OCH_3_	H	80%

**Table 2:** IC_50s_ of **6b** against Hs766T, HPAF-II, PDAC3, and PATU-T cells.

IC_50_ ^a^ (µM) ± SEM		
Compound	Cell Line	IC_50_ ± SEM
**6b**	Hs766T	5.7 ± 0.60
	PDAC3	6.9 ± 0.25
	HPAF-II	9.8 ± 0.70
	PATU-T	10.7 ± 0.16

^a^ Values (in µM) are reported as means ± SEM (Standard Error of the Mean) of three separate experiments.

**Table 3:** ADME predictions of compound **6b**.

Parameters	Score
n. H-bond acceptor	6
n. H-bond donor	0
n. rotable bonds	3
LogPo/w(iLOGP)	3.67
Lipinski’s rule violation	No
Bioavailability score	0.55
GI absorption	High
BBB permeation	no

## Data Availability

Not applicable.
